# *Trioxys liui* Chou & Chou, 1993 (Hymenoptera, Braconidae, Aphidiinae): an invasive aphid parasitoid attacking invasive *Takecallis* species (Hemiptera, Aphididae) in the Iberian Peninsula

**DOI:** 10.3897/zookeys.944.51395

**Published:** 2020-06-30

**Authors:** Ehsan Rakhshani, Jose Michelena Saval, Nicolas Pérez Hidalgo, Xavier Pons, Nickolas G. Kavallieratos, Petr Starý

**Affiliations:** 1 Department of Plant Protection, College of Agriculture, University of Zabol, Zabol, 98613–35856, IR Iran University of Zabol Zabol Iran; 2 Cavanilles Institute of Biodiversity and Evolutionary Biology (ICBiBE), Universitat de València, 46980, València, Spain Universitat de València València Spain; 3 Instituto de Biología Integrativa de Sistemas (I2SysBio), Universidad de València-CSIC, 46071, València, Spain Universidad de València-CSIC Valencia Spain; 4 Universitat de Lleida, Department of Crop and Forest Sciences, Agrotecnio Centre, Rovira Roure 191, 25198, Lleida, Spain Universitat de Lleida Lleida Spain; 5 Laboratory of Agricultural Zoology and Entomology, Department of Crop Science, Agricultural University of Athens, 75 Iera Odos str., 11855, Athens, Attica, Greece Agricultural University of Athens Athens Greece; 6 Laboratory of Aphidology, Institute of Entomology, Biology Center AVCR, Branišovská 31, 370 05, České Budějovice, Czech Republic Institute of Entomology, České Budějovicé Czech Republic

**Keywords:** Bamboo, invasive species, new association, parasitoid

## Abstract

Biological invasion of aphids and other insects has been increased due to long distance commercial transportation of plant material. The bamboo-aphid-parasitoid association is strictly specific and even though it does not develop interactions with the local environment it should be listed as part of the fauna of southwestern Europe. On-going research regarding aphids and their aphidiine parasitoids in Spain has yielded a new association of *Trioxys liui* Chou & Chou, 1993 with an undescribed species of *Takecallis* aphids on bamboo, *Phyllostachys* spp. Here we present the first association of *T. liui* with aphids of the genus *Takecallis* that attack bamboos. *Trioxys liui* is known as a parasitoid of *Cranaphis formosana* (Takahashi, 1924) and *Phyllaphoides bambusicola* Takahashi, 1921 on bamboos in China and Russia. The accidental introduction of this parasitoid species to southwestern Europe has been probably realized through transportation of contaminated bamboo plant material. In the current study, a new host association is recorded for *T. liui*. Its potential to invade other bamboo-associated aphids and the significance of the tritrophic bamboo-aphid-parasitoid interactions in the new environments are also discussed.

## Introduction

Bamboo is a common name that encompasses at least 1250 species and 75 plant genera (Scurlock et al. 2000) within the family Poaceae (subfamily Bambusoideae). Although bamboos are distributed mostly in the tropics, they also naturally occur in the subtropical and temperate zones of all continents except Europe (Ohrnberger 2002). China (Qiu et al. 1992) and India (Shanmughavel and Francis 1996) are regions where the highest number of bamboo species are growing, mainly as natural stands. On the other hand, species of the genera *Phyllostachys* and *Pleioblastus* are ornamental plants that have been introduced into Europe for commercial purposes (Schilberszky 1911). Furthermore, there is an increasing interest in the use of bamboos as energy plants (Scurlock et al. 2000; Wright 2006; Potters et al. 2009) and for other industrial applications (van der Lugt et al. 2006; Lipp-Symonowicz et al. 2011). The well known group of aphids that belongs to genus *Takecallis* Mastumura is associated with bamboos. These aphids have been considered as invasive organisms in different parts of the world where bamboos are purposefully or accidentally introduced (Stroyan 1964; Coffelt and Schultz 1990; Limonta 1990; Lazzari et al. 1999; Trejo-Loyo et al. 2004; Valenzuela et al. 2010). Three invasive *Takecallis* species, i.e., *Takecallis arundinariae* (Essig, 1917), *Takecallis taiwanus* (Takahashi, 1926) and *Takecallis arundicolens* (Clarke, 1903) (Calaphidinae, Panaphidini) have already been recorded in several European countries (see Rakhshani et al. 2017). It should be noted that the three aforementioned aphid species occur in Spain (Nieto Nafría and Mier Durante 1998; Suay-Cano and González-Funes 1998; Pons and Lumbierres 2004).

A new parasitoid species, *Trioxys remaudierei* Starý & Rakhshani, 2017 (Hymenoptera, Braconidae, Aphidiinae), has been recently described and associated with two bamboo aphids, *T. arundinariae* and *T. taiwanus* in France and Spain (Rakhshani et al. 2017). This evidence led to an exhaustive investigation for parasitoids of bamboo aphids in southwestern Europe that resulted in detection of a *Trioxys* species emerging from *Takecallis* aphids, which infest bamboo groves in Spain. Surprisingly, this parasitoid species was not conspecific with *T. remaudierei*. Here we determine and illustrate a new aphid parasitoid association from bamboos in Spain. An annotated world-review of *Takecallis* aphids attacking bamboos is also provided.

## Material and methods

**Figure 1.**
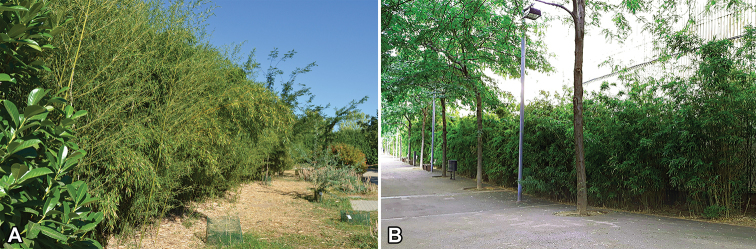
General scheme of the sampling localities in the Iberian Peninsula **A** patch of *Phyllostachys aurea* in the Arboretum of Lleida **B** street garden with rows of bamboo (*Phyllostachis* sp.) in Barcelona.

Research on bamboo aphids was carried out in the east and northeast of the Iberian Peninsula: Valencia, Barcelona and Lleida (Fig. 1). In Valencia, samples were collected from the Botanical Garden of the University of Valencia during spring 2017 (March to June). All species of bamboos that are growing in the Botanical Garden were sampled: *Bambusa ventricosa*, *Dendrocalamus giganteus*, *Phyllostachys nigra*, *Phyllostachys viridis*, *Phyllostachys aurea*, *Pleioblastus linearis*, *Pleioblastus pumilus*, *Pleioblastus pygmaeus* and *Shibataea kumasasa*. The aphid colonies were inspected in the field and the laboratory so as to be checked for any occurence of parasitoids. At the begining of April (10.iv.2017), several mummies of *Takecallis* spp. were recorded on mixed colonies of the three species of *Takecallis* present in the Iberian Peninsula (*T. arundicolens*, *T. arundinariae* and *T. taiwanus*). Samples from Barcelona originated from two locations: 1) a street garden (41°24'24.15"N, 2°11'49.78"E, 5 m a.s.l.), where a 100 m × 2 m row of bamboo (Phyllostachys nr. aurea) are planted; 2) the gardens of the Royal Palace of Pedralbes, where different irregular size patches of bamboo (mainly Phyllostachys nr. aurea), of a whole area of about 300 m^2^ are planted (41°23'16.05"N, 2°07'03.53"E, 99 m a.s.l.). Both locations are separated by a 7 km straight line. Visual inspection determined the presence of some common aphid species on bamboo (*Melanaphis bambusae* (Fullaway, 1910) (Aphidinae, Aphidini); *T. taiwanus* and *T. arundinariae*), but the plants from the first location were mostly infested by *Takecallis* species. Mummies and live aphids within isolated or mixed colonies of *T. taiwanus* and *T. arundinariae* were collected in May and June 2018. In Lleida, samples from *Indocalamus tessellatus* and *P. aurea* were collected from the Arboretum and Botanical Garden Pius Font i Quer (41°37'29.96"N, 0°36'11.70"E, 182 m a.s.l.). *Phyllostachys aurea* was the dominant bamboo species there, occupying most of the bamboo plantation area (700 m^2^), but there were also small patches of other bamboos species such as *Phyllostachys aureosulcata*, *P. nigra*, *Pleiobalastus fortunei*, *Pseudosasa usawae*, *S. kumasasa*, *I. tessellatus* and *Fargesia scabrida*. Aphids from the genus *Takecallis* were found on *P. aurea* and *I. tessellatus*.

Aphid colonies, containing both alive individuals and mummies, were sampled with small pieces of bamboo plants that were gently cut with scissors. Samples were transferrred to the laboratory, where they were maintained at room temperature. Some adult aphids were preserved in a solution containing two parts of 90% ethanol to one part of 75% lactic acid (Eastop and van Emden 1972) for identification. Then, they were compared with the keyed material according to Nieto Nafría and Mier Durante (1998) and Blackman and Eastop (2019). Aphid nomenclature and classification follows Favret (2019). The emerged parasitoids were captured with an aspirator and directly dropped into 70% ethanol. The external morphology of parasitoids was studied using a Nikon Eclips E200 stereomicroscope (Nikon Corp., Japan). The parasitoid specimens were identified according to Chen and Shi (2001) and Davidian (2005). Illustrations were traced on the digital photographs captured from the slides with a Canon EOS 700D (Canon Inc., Japan) in Adobe Illustrator CS5 and were processed in Photoshop CS5 (Adobe systems Inc., San Jose, USA).

A series of voucher specimens of the emerged aphid parasitoids was sorted and preserved in absolute ethanol, kept in a refrigerator for DNA extraction. Total DNA was extracted separately from two individuals (a male and a female) following the HotSHOT method (Truett et al. 2000) using 60 µl of both alkaline lysis and neutralizing reagents. A 710 bp fragment of the 5' region of the mitochondrial gene coding the cytochrome c oxidase subunit 1 (COI) was sequenced, using the primer pair LCO1490 and HCO2198 as described by Folmer et al. (1994). PCR and sequencing procedures are outlined in Pérez Hidalgo et al. (2012). After removing sequences corresponding to primers used in the PCR reaction, the sequences obtained from each sample consisted of 658 nucleotides. DNA sequences of both male (a) and female (b) parasitoids were deposited in the GenBank database under the accession numbers MT324250 and MT324249, respectively. The existing mtCOI sequences in NCBI for *Trioxys* and *Binodoxys* spp. were retrieved and aligned in MEGA X (Kumar et al. 2018) with the integrated ClustalW using default parameters. The evolutionary history was inferred using the Neighbor-Joining method (Saitou and Nei 1987) with pairwise deletion of missing sites and Kimura-2-Parameter (K2P) distances (Kimura 1980). The optimal tree with the sum of branch length = 1.48087269 is shown. The percentage of replicate trees in which the associated taxa clustered together in the bootstrap test (1000 replicates) are shown next to the branches. *Ephedrus persicae* Froggatt, 1904 (Hymenoptera, Braconidae, Aphidiinae – KY213710.1) and *Praon volucre* (Haliday, 1833) (Hymenoptera, Braconidae, Aphidiinae – KJ698515.1) were used as outgroups for the molecular analysis. The tree is drawn to scale, with branch lengths in the same units as those of the evolutionary distances used to infer the phylogenetic tree. The analysis involved 34 nucleotide sequences. Codon positions included were 1st+2nd+3rd+Noncoding. All ambiguous positions were removed for each sequence pair (pairwise deletion option). There were a total of 519 positions in the final dataset.

The material is deposited in the Entomology Collection of the University of Valencia of the Cavanilles Institute of Biodiversity and Evolutionary Biology, in the Laboratory of Entomology of the Department of Crop and Forest Sciences of the University of Lleida, in the collection of P. Starý (Academy of Sciences of the Czech Republic), in the Laboratory of Agricultural Zoology and Entomology of the Agricultural University of Athens and in the collection of Department of Plant Protection, University of Zabol.

## Results

**Figure 2.**
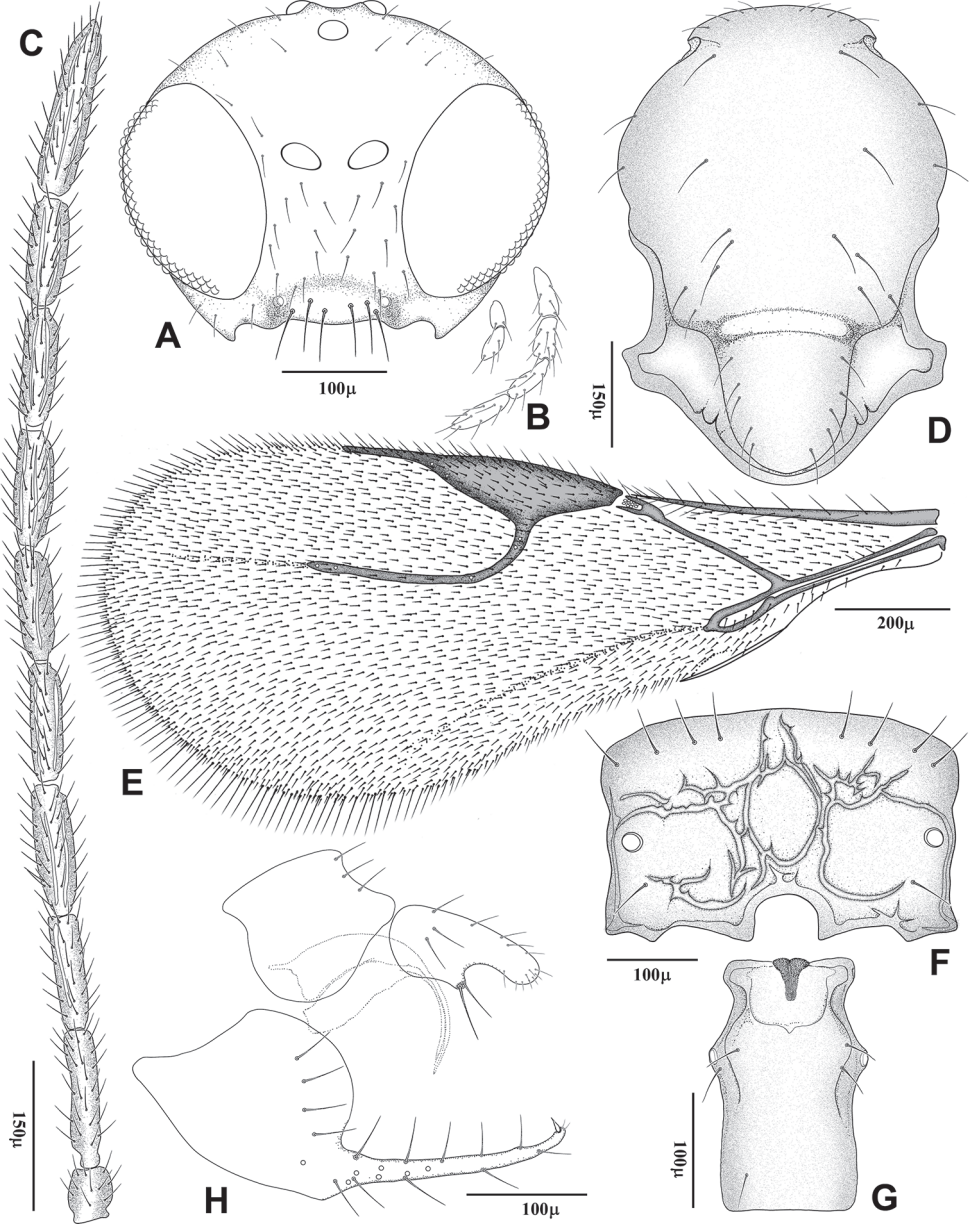
*Trioxys liui* – female **A** head, frontal view **B** maxillary and labial palps **C** antenna **D** mesonotum and scutellum, dorsal view **E** forewing **F** propodeum **G** petiole, dorsal view **H** genitalia, lateral aspect.

**Figure 3.**
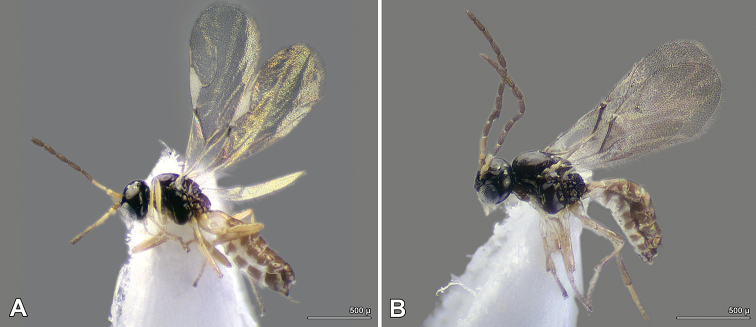
*Trioxys liui* – general habitus **A** female **B** male.

Four aphid species are present in the Botanical Garden of the University of Valencia living in mixed colonies only on *P. viridis*, *P. aurea*, and *P. linearis*. Three of them, which are not attended by ants, belong to the genus *Takecallis* (*T. arundicolens*, *T. arundinariae*, *Takecallis* sp. (probably a new species)). However, the fourth of these species, *M. bambusae*, was strongly attended by the ant *Lasius grandis* Forel, 1909 (Hymenoptera, Formicidae). Out of all parasitoid individuals emerged from 20 mummies of *Takecallis* sp. collected from Valencia we obtained six males and six females of an aphidiine whose morphological characters clearly matched those of *T. liui*. Samples collected from Barcelona and Lleida led to the emergence of additional 11 female and 13 male specimens of *Trioxys liui* Chou & Chou (Hymenoptera, Braconidae, Aphidiinae) originating from *T. taiwanus* and *T. arundinariae* which are reported below. Although *T. liui* has been originally figured (Chou and Chou 1993) and later keyed by Chen and Shi (2001) and Davidian (2005, 2007), additional illustrations (Figs 2, 3) are provided to increase the taxonomical evidence for comparison with other parasitoid species of bamboo aphids.

### Trioxys liui

Taxon classificationAnimaliaHymenopteraBraconidae

Chou & Chou, 1993

1BEDB033-2B86-5465-A406-F975A294B43C

Figures 2 , 3

#### Material examined.

6♀ 6♂, Spain: Valencia, Botanical Garden, 39°28'36.6"N, 0°23'09.1"W, 17 m a.s.l., collected from 15.iv.2018 to 6.vi.2018, ex *Takecallis* sp. on *Phyllostachys aurea*, J.M. Michelena leg.; 2♀, Barcelona, public garden, 41°24'24.25"N, 2°11'49.78"E, 2 m a.s.l., 06.v.2018, ex *Takecallis taiwana* (Takahashi) on *Phyllostachys* sp., X. Pons leg. [Sample B-1052]; 2♀ 4♂, Barcelona, public garden, 41°24'24.15"N, 2°11'49.78"E, 2 m a.s.l., 18.v.2018, ex *Takecallis taiwana* (Takahashi) on *Phyllostachys* sp., X. Pons leg. [Sample B-1054]; 2♀ 4♂, same collecting data as for preceding, 06.vi.2018, ex *Takecallis arundinariae* (Essig) on *Phyllostachys* sp., X. Pons leg. [Sample B-1064]; 3♀, same collecting data as for preceding, ex *Takecallis taiwana* (Takahashi) on *Phyllostachys* sp., X. Pons leg. [Sample B-1065]; 2♀ 1♂, same collecting data as for preceding, captured on *Phyllostachys* sp., X. Pons leg. [Samples B-1064 + B-1065]; 1♂, Barcelona, public park, 41°23'16.05"N, 2°07'03.53"E, 71 m a.s.l., 15.vi.2018, captured on *Phyllostachys* sp., X. Pons leg. [Sample B-1070]; 1♂, Lleida Arboretum, 41°37'29.96"N, 0°36'11.70"E, 181 m a.s.l., 24.v.2018, captured on *Indocalamus tessellatus*, X. Pons leg. [Sample L-1059].

#### Morphological diagnosis.

**Female – *Body length***: 1.4–1.6 mm, forewing length 1.5–1.6 mm. ***Clypeus*** (Fig. 2A) narrow with 6 long setae on dorsal surface. Maxillary palp with 4 palpomeres, labial palp with 2 palpomeres (Fig. 2B). ***Antenna*** (Fig. 2C) filiform, with 11 antennomerese, covered mainly with semierect setae, slightly shorter than the diameter of segments. Flagellomere 1 (F1) 1.15–1.22× as long as F2 and 3.60–3.75× as long as maximally wide. F1 and F2 with 1–2 and 1–3 longitudinal placodes, respectively. ***Mesoscutum*** (Fig. 2D) smooth with notaulices hardly visible at anterior part, sparsely setose. ***Forewing*** (Fig. 2E) stigma elongate, triangular with slightly convex outline, 2.90–3.10× as long as wide and 3.00–3.20× as long as R1. Vein r&RS extended beyond R1. Wing margin with very long frings. ***Propodeum*** (Fig. 2F) with well developed carinae, irregularely branched at anterior and lateral parts. Central areola partially divided by irregular internal carinae. Upper and lower parts of propodeum with 8 and 2 long erected setae, respectively. ***Petiole*** (Fig. 2G) short, 1.55–1.80× as long as wide at spiracles with a pair of long setae near each prominent spiracular tubercle and a single seta at posterio-dorsal area. ***Ovipositor sheath*** (Fig. 2H) stout, with smooth dorsal outline, deeply concaved ventrally in anterior edge, sharly expanded into a ventral projection bearing a pair of long setae. Prongs distinctly separated at base, almost stright, progressively constricted and upcurved at apex. Ventral side of prongs bear 4–5 prependicular setae, with single claw-shape seta and pair of short setae at apex.

***Color*** (Fig. 3A). Head and mesosoma dark brown to black, gaster brown. Antenna brown, mouth parts, pedicel, F1, legs and petiole yellowish brown. Wings infumated. Apex of oviposirot sheath dark brown.

**Male** (Fig. 3B) – Antennae with 13 antennomeres, body length 1.4–1.5 mm.

#### Molecular data.

**Figure 4.**
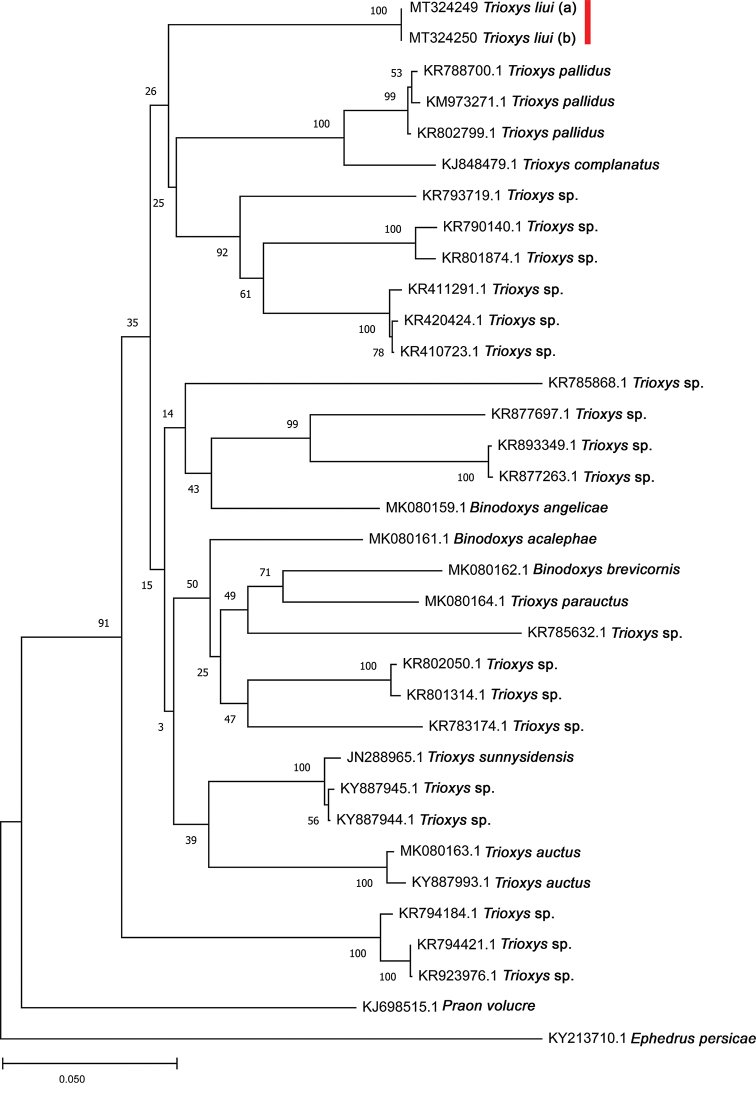
Neighbor-Joining tree based on the partial mtCOI sequences from *Trioxys* and *Binodoxys* spp., including *Trioxys liui*, with *Praon volucre* and *Ephedrus persicae* as outgroups (NCBI accession no). Numbers next to nodes are the bootstrap values.

The DNA sequences of the mtCOI gene were obtained from a single specimen of both male and female of *T. liui*, with no intraspecific genetic distance (0.0%). The interspecific genetic distances of mtCOI within *Trioxys* species ranged from 10.9% to 14.7%. The uncorrected pairwise genetic distances (p-distance) of mtCOI, separated *T. liui* from all other *Trioxys* species, with at least 10% of the sequence divergence (Table 1). After the reconstruction of the Neighbor-Joining tree, *T. liui* stands basal to the branch that includes *Trioxys pallidus* (Haliday, 1833) and *Trioxys complanatus* Quilis, 1931 (Fig. 4). No further analysis was possible because almost all deposited sequences originated from specimens that were identified only at generic level.

**Table 1.** Uncorrected pairwise genetic distances between *Trioxys liui* and other *Trioxys*/ *Binodoxys* species based on mtCOI sequences resulted from MEGA X.

	Species	Accession No.	1	2	3	4	5	6	7	8	9	10
**1**	*T. liui*	MT324249										
**2**	*T. pallidus*	KM973271.1	0.139									
**3**	*T. complanatus*	KJ848479.1	0.147	0.052								
**4**	*Trioxys* sp.	KR411291.1	0.112	0.137	0.134							
**5**	*Trioxys* sp.	KR420424.1	0.109	0.134	0.134	0.005						
**6**	*T. auctus*	KY887993.1	0.133	0.145	0.136	0.141	0.138					
**7**	*T. parauctus*	MK080164.1	0.163	0.128	0.153	0.136	0.136	0.125				
**8**	*T. sunnysidensis*	JN288965.1	0.125	0.137	0.136	0.128	0.128	0.083	0.086			
**9**	*B. brevicornis*	MK080162.1	0.144	0.153	0.158	0.133	0.133	0.135	0.133	0.122		
**10**	*B. acalephae*	MK080161.1	0.122	0.128	0.128	0.131	0.128	0.104	0.104	0.101	0.101	
**11**	*B. angelicae*	MK080159.1	0.128	0.143	0.151	0.137	0.134	0.127	0.130	0.081	0.112	0.109

## Discussion

*Trioxys liui* is an aphidiine parasitoid of two bamboo aphids in China: *Cranaphis formosana* (Takahashi, 1924) (Liu 1975) and *Phyllaphoides bambusicola* Takahashi, 1921 (Calaphidinae, Panaphidini) on *Phyllostachys makinoi* (Chou and Chou 1993). So far, *T. arundinariae* in Spain and *T. taiwanus* in France, both attacking *Phyllostachys* spp., have been reported as the only hosts of the newly described aphidiine parasitoid, *T. remaudierei* in Western Europe (Rakhshani et al. 2017). Our findings add *T. liui* as a new member of the parasitoid fauna of Spain that parasitizes even a new species of *Takecallis* (Valencia). This evidence contributes both to the increase in the number of exotic parasitoid species on bamboo aphids and the potential of interspecific relations of parasitoids. The new association, *T. liui/ Takecallis* spp. (Valencia, Barcelona and Lleida) is also a new assemblage apart from *T. remaudierei/ Takecallis* spp. (Paris-France and Lleida-Spain). Therefore, our results clearly document the invasion and/or subsequent adaptation of the southeastern Asian endemic species into Western Europe. The lack of information on *T. liui*, with the exception of the knowledge about its original region, China, has indicated a direct interaction of southeastern Asia with Spain through bamboo plant material, that accidentally included aphid and parasitoid contamination. Furthermore, secondary exchanges of shipments between the gardeners are also considered possible. However, similar evidence from insects in other areas has not been documented yet. It is noteworthy to emphasize the obvious morphological differences between the two parasitoids of *Takecallis* in Europe, which might reveal new bamboo-aphid-parasitoid associations. *Trioxys remaudierei* has long ventral prongs fused over two-thirds of their length. However, prongs of *T. liui* are short and completely separated. Among European species, *Trioxys betulae* Marshall, 1896 has also partially fused prongs. However, this parasitoid has a different host range since it includes aphid species that do not belong to genus *Takecallis* (Rakhshani et al. 2017). Despite the complexity of the taxonomy of the genus *Trioxys*, little attention has been devoted to using molecular data for identification and resolving questions on their classification (mainly unpublished). None of these efforts include the parasitoids of *Takecallis* aphids.

Generally, bamboos grow and spread fast (Cao et al. 2011), a trait that can crowd out native plant species. The allelopathic secretions of various bamboo species can also prohibit the growth of the nearby plant species (Chou and Yang 1982; Chou 1999; Rawat et al. 2017). However, ornamental bamboos should be protected against invasive herbivorous insects that could be distributed along with their host plant material in botanical gardens in Europe. With few exceptions (Potenza 2005), there is no record of aphid contamination hazards on bamboos, but their associations should be considered and listed in the Iberian Peninsula. The above mentioned case was the infestation of bamboo hedges by *T. taiwanus*, which were successfully controlled by the application of chemical pesticides.

Aphids of the genus *Takecallis* and their parasitoids are strictly associated with bamboo while any other interactions with the environmental local fauna have not been determined yet (Rakhshani et al. 2017). Therefore, in terms of the specific associations of *Takecallis* spp. and their parasitoids on bamboos, no hazardous effects are expected on the environment that is reserved for their plantation (i.e., arboretums, parks, public and private gardens). Although genus *Takecallis* is represented by seven valid species worldwide (Table 2), a vast number of aphid genera are recorded in association with bamboos and other plant species (e.g., Anacardiaceae, Cyperaceae, Poaceae, Rosaceae, Styracacee) (Blackman and Eastop 1994, 2006). Apart from aphids, a rich fauna of insects is also associated with bamboos in their area of origin (Revathi and Remadevi 2011).

**Table 2.** *Takecallis* species associated with various bamboos and their distribution.

**Aphid species**	**Distribution**	**Host plant**	**References**
*Takecallis affinis* Ghosh, 1986	India	*Bambusa* sp.	Ghosh 1986
		*Chimonobambusa jaunsarensis*	
*Takecallis alba* Lee, 2018	South Korea	*Pseudosasa* sp.	Lee and Lee 2018
		*Sasa* spp.	
*Takecallis arundicolens* (Clarke, 1903)	China, Eastern Russia, Europe (Spain, France, Netherlands, Serbia), Japan, Korea, North America, Taiwan	*Arundinaria japonica*	Leclant 1966;
		*Bambusa* sp.	Higuchi 1972;
		*Phyllostachys* sp.	Lampel and Meier 2003;
		*Pleioblastus chino*	Qiao and Zhang 2004;
		*Pseudosasa japonica*	Pons and Lumbierres 2004;
		*Sasa nipponica*	Blackman and Eastop 2019;
		*Sasa palmate*	Piron 2009;
		*Sasa paniculata*	Lee and Lee 2018;
		*Sasa senaanensis*	Petrović-Obradović et al. 2018
		*Sasaella ramosa*	
*Takecallis arundinariae* (Essig, 1917)	Australia, Central America (Mexico), China, Eastern Russia, Europe (Greece, Hungary, Italy, Netherlands, Portugal, Spain, Switzerland, United Kingdom), India, Japan, Korea, North America, South America (Brazil), Taiwan	*Arundinaria graminea*	Higuchi 1968;
		*Arundinaria japonica*	Higuchi 1972;
		*Bambusa bambos*	Raychaudhuri 1973;
		*Bambusa rigida*	Ghosh 1980;
		*Bambusa stenostach*	Ghosh and Quednau 1990;
		*Bambusa textilis*	Coffelt and Schultz 1990;
		*Dendrocalamus asper*	Giacalone and Lampel 1996;
		*Phyllostachys aurea*	Aguiar and Ilharco 1997;
		*Phyllostachys bambusoides*	Nieto Nafría and Mier Durante 1998;
		*Phyllostachys castillonis*	Suay-Cano and González-Funes 1998;
		*Phyllostachys dulcis*	Lazzari et al. 1999;
		*Phyllostachys edulis*	Qiao and Zhang 2004
		*Phyllostachys iridescens*	Trejo-Loyo et al. 2004;
		*Phyllostachys mannii*	Pons and Lumbierres 2004;
		*Phyllostachys puberula*	Cœur d’acier et al. 2010;
		*Phyllostachys viridiglaucescens*	Blackman and Eastop 2019;
		*Pseudosasa japonica*	Barbagallo and Ortu 2009;
		*Sasa nipponica*	Piron 2009;
		*Sasa palmate*	Valenzuela et al. 2010;
		*Sasa senaanensis*	Basky and Neményi 2014;
		*Sinoarundinaria niitakayamensis*	Lee and Lee 2018
		*Sinobambusa tootsik*	
*Takecallis assumentus* Qiao & Zhang, 2004	China	*Bambusa* sp.	Qiao and Zhang 2004
*Takecallis himalayensis* Chakrabarti, 1988	India	*Arundinaria jounsarensis*	Chakrabarti 1988;
		*Bambusa* sp.	Ghosh and Quednau 1990
*Takecallis sasae* (Matsumura, 1917)	Japan	*Phyllostachys* sp.	Higuchi 1968, 1972
		*Pleioblastus* sp.	
		*Sasa nipponica*	
		*Sasa paniculata*	
*Takecallis taiwanus* (Takahashi, 1926)	Central America (Mexico), China, Europe (Hungary, Croatia, Netherlands, Spain, United Kingdom, Russia), Georgia, Japan, Korea, New Zealand, North America, South Africa, South America (Argentina, Brazil, Chile), Taiwan	*Arundinaria anceps*	Stroyan 1964; Higuchi 1968; Higuchi 1972;
		*Arundinaria gigantea*	Giacalone and Lampel 1996;
		*Bambusa stenostach*	Nieto Nafría and Mier Durante 1998;
		*Dendrocalamus asper*	Suay-Cano and González-Funes 1998;
		*Phyllostachys arcana*	Foureaux and Kato 1999;
		*Phyllostachys aurea*	Lazzari et al. 1999;
		*Phyllostachys bambusoides*	Gonzales et al. 2000;
		*Phyllostachys castukkinis*	Peronti and Sousa-Silva 2002;
		*Phyllostachys dulcis*	Qiao and Zhang 2004;
		*Phyllostachys nigra*	Pons and Lumbierres 2004;
		*Phyllostachys sulphurea*	Trejo-Loyo et al. 2004;
		*Phyllostachys viridiglaucescens*	Ortego et al. 2004;
		*Pleioblastus amarus*	Blakman and Eastop 2019;
		*Pleioblaslus variegatus*	Ripka 2008; Simala et al. 2008;
		*Sasa* spp.	Maslyakov and Izhevsky 2011;
		*Shibataea kumasasa*	Lee and Lee 2018

## Conclusion

The increasing attention of bamboos from both ornamental and industrial aspects will evidently lead to the invasion of more alien species into Europe and other parts of the world. A concrete knowledge on the status of those insects in southeastern Asia needs to be elaborated, which is the current bamboo source to Europe. Phytosanitary authorities should very carefully examine all imported bamboo material at any entry point of Europe to intercept alien insect species that could be a potential threat to the local plantations and entomofauna.

## Supplementary Material

XML Treatment for Trioxys liui
